# Using a Geographical Information System to investigate the relationship between reported cryptosporidiosis and water supply

**DOI:** 10.1186/1476-072X-3-15

**Published:** 2004-07-23

**Authors:** Sara Hughes, Qutub Syed, Sarah Woodhouse, Iain Lake, Keith Osborn, Rachel M Chalmers, Paul R Hunter

**Affiliations:** 1 Health Protection Agency Northwest, Chester, UK; 2 School of Environmental Sciences, University of East Anglia, Norwich, UK; 3 United Utilities, Warrington, UK; 4 HPA Cryptosporidium Reference Unit, Swansea, UK; 5 School of Medicine, Health Policy and Practice, University of East Anglia, Norwich, UK

## Abstract

**Background:**

This paper reports on a study investigating the epidemiology of sporadic cryptosporidiosis in the North West of England and Wales using a Geographical Information System (GIS) to map location of residence of cases. Some 747 reports of cases were made to CDSC North West of which 649 reports were suitable for analysis. Cases were plotted on the maps of water supply zone and water quality area boundaries, provided by the two main water utilities.

**Results:**

It was notable that there were major spatial variations in attack rate across the North West and Wales. The most dramatic example was the large difference between the Greater Manchester conurbation with many reports and Liverpool with none. Given the distribution of previously detected waterborne outbreaks in the region it was initially thought that drinking water source may be an explanation. However, an analysis of the distribution of cases in the Greater Manchester area showed no correlation with any of five water supplies that serve the conurbation.

**Conclusions:**

Our study has shown a dramatic variation in the incidence of laboratory confirmed cryptosporidiosis within two regions of the United Kingdom. Further analysis has not been able to prove drinking water as a likely explanation of this variation which so far remains unexplained.

## Background

Cryptosporidiosis is infection with species of the genus *Cryptosporidium*. Most infections in the UK are due either to *C. parvum *(previously known as *C. parvum *genotype 2 or bovine strain) or *C. hominis *(previously known as *C. parvum *genotype 1 or human strain) [1]. *Cryptosporidium *spp. are protozoan parasites. In otherwise healthy individuals they tend to cause a self-limiting form of gastroenteritis which can last for several days and, sometimes, weeks. In patients with certain forms of immune deficiency, most notable the Acquired Immune Deficiency Syndrome (AIDS), the infection can cause a severe and prolonged diarrhoeal illness which, prior to the widespread use of at highly effective antiretroviral therapy was often fatal [2]

Cryptosporidiosis has now become the most commonly identified protozoal cause of gastroenteritis in the United Kingdom. Most of the epidemiological data to-date has been related to reports of outbreaks. Between the years 1983 to 1997 there were 80 outbreaks cryptosporidiosis in England and Wales affecting 4649 individuals [3]. Of these 80 outbreaks, 25 affecting 3455 cases were associated with drinking water. Indeed large outbreaks of cryptosporidiois have often been associated with drinking water [4,5]. Outbreaks of cryptosporidiosis were a particular problem in the North West Region of England during the 1990s where a single unfiltered surface water source was responsible for several outbreaks [2]. However, outbreak-related cases represent only a small proportion (<10%) of the total cases reported to national surveillance.

The epidemiology of sporadic (non-outbreak-related) cases is largely unknown. Of three large case-control studies reported in the past few years only one found an association with drinking mains water [7-9]. The study that did find an association with drinking water was undertaken in a largely rural area that when the study was undertaken received some of its water from systems that did not have modern filtration plants. The other two studies identified contact with another case, contact with cattle and overseas travel as being the main risk factors for sporadic infection.

The question remains what proportion of sporadic cryptosporidiosis infections may be due to the consumption of mains drinking water. This paper reports a study using GIS to investigate the epidemiology of sporadic cryptosporidiosis and particularly address the issues of whether sporadic cases are also associated with consumption of drinking water.

## Results

**Figure 1:**
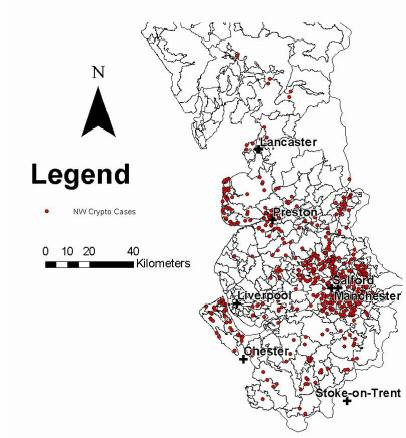
Cryptosporidiosis cases in the North West, January 2001 to February 2002

**Figure 2:**
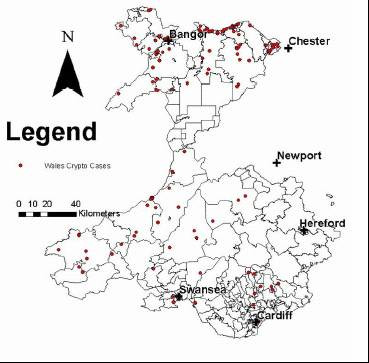
Cryptosporidiosis cases in the Welsh Water Area, February 2001 to February 2002

**Figure 3:**
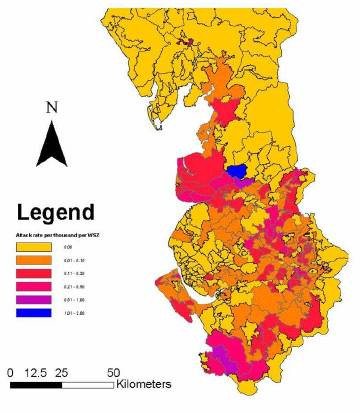
**Cryptosporidium attack rates in the North West. **Attack rates calculated for each Water Supply Zone in the North West, January 2001 to February 2002 (per 1000 population).

Table 1 shows the number of records from each health authority and the number of exclusions, including reasons for exclusion. Figures 1 and 2 show the geographical distribution of individual cases by indicating a dot on the map of the water supply zones (water quality area for Wales). Figure 3 indicates the attack rates for each zone in the North West where the shading indicates a range of attack rates. Care should be taken in interpreting the zone rates as the populations covered by each zone/area varied substantially. In some zones high attack rates were seen despite only a single case being identified because of a low denominator population. The area specific attack rates are not shown for Wales as numbers of cases was smaller.

**Table 1:** Health authority of reported cases, including reasons for exclusion from analysis

				Reasons for excluding post codes				
					
Health Authority	Total records	Included in analysis	Excluded	Incorrect	Duplicate	Incomplete	Missing	% excluded
Bro Taf	2		2	2				100
Bury and Rochdale	51	43	8	4	2	1	1	15.7
Dyfed Powys	49	44	5	5				10.2
East Lancashire	47	45	2	2				4.3
Gwent	12	12	0					0
Iechyd Morgannwg	6		6	6				100
Morecambe Bay	13	10	3			3		23.1
Manchester	69	48	21	3		10	8	30.4
N Cheshire	6	6	0					0
North Wales	121	111	10	9		1		8.3
North West Lancashire	74	59	15	3	2		10	20.3
South Cheshire	63	63	0					0
South Lancashire	20	20	0					0
Salford	34	28	6			4	2	17.6
St Helens & Knowsley	8	8	0					0
Stockport	66	60	6	1	1		4	9.1
West Pennine	32	29	3		1	1	1	9.4
Wigan and Bolton	46	35	11	2	4	4	1	23.9
Wirral	28	28	0					0
TOTAL	747	649	98	37	10	24	27	13.1

It can be seen that there is substantial spatial variation in the distribution of reported cases. In part, this variation can be explained by variation in population density. However, much of the variation is unexplained. For example, reports from Liverpool are very uncommon, whilst reports from Greater Manchester are very common.

It was decided to investigate the excess case reporting from Greater Manchester in further detail to look for any possible association with water supplies. Water to the Greater Manchester area comes from five main water treatment works; Lostock (derived from Thirlmere in the Lake District and chlorinated but not filtered), Woodgate Hill (derived from Haweswater and Windermere via the Watchgate Treatment Works near Kendal where the water is treated by rapid gravity sand filtration, though not chemically coagulated before spring 2003), Arnfield-Godley (chemical coagulation, clarification and rapid gravity sand filtration), Buckton Castle (chemical coagulation, dissolved air flotation and rapid gravity sand filtration) and Wybersley (chemical coagulation, dissolved air flotation and rapid gravity sand filtration).

**Figure 4:**
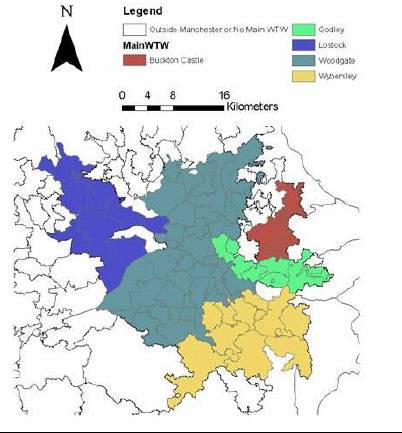
**Dominant water treatment works in Greater Manchester during 2001. **Each water supply zone in the Greater Manchester area is identified and colour coded to illustrate which of the five water treatment works supply most of the drinking water. Where there is no single dominant source the zone is left uncoloured

In order to determine whether there was any relationship between attack rate and water supply, all water supply zones in the North West that received any water from one or more of these five supplies were identified (Figure 4). For each of these water supply zones, the proportion of the supply from each treatment works were was obtained from United Utilities. The correlation between the attack rate and proportion of water from each treatment works was tested using Kendall's rank correlation (Table 2) [10]. The figure adjusted for ties was used. There was no significant correlation between water source and attack rate.

**Table 2:** Water supply and cases of Cryptosporidiosis Correlation between water supply zone specific attack rate and proportion of water received from each of the five main water treatment works supplying Greater Manchester.

Water treatment works	Z	P value
Lostock	-1.084	0.2782
Woodgate Hill	1.713	0.0867
Arnfield – Godley	-1.186	0.2353
Buckton Castle	-0.628	0.5294
Wybersley	0.451	0.6517

## Discussion

As already mentioned, care should be taken in the interpretation of this analysis. It is notable that the proportion of reports that could not be allocated a correct postcode varied from one health authority to another to some extent. Also variation in attack rate between water supply zones or water quality areas was as likely to be due to differences in population size as to differences in reported cases. This was most obvious in zones/areas with relatively small population sizes where random effects could have a particularly important affect. However, there are a number of obvious features.

The most obvious is the large number of cases from the Greater Manchester conurbation. This covered the Bury and Rochdale, Manchester, Salford, Stockport, West Pennine, and Wigan and Bolton Health Authorities. This excess of cases in Manchester is even more remarkable when compared with the virtual absence of cases from the Liverpool conurbation (Liverpool, Sefton and St Helen's & Knowsley Health Authorities). The reason for the excess of cases in Greater Manchester is unclear. Although different reporting habits could play a part, we doubt that it could explain more than a small part of the difference. Reporting practices are not that greatly different across the North West [10]. A sero-epidemiological study, currently underway, may be able to determine whether the low reporting rate from Liverpool is real or not.

An alternate explanation could be that the increase represents different water supplies. Salford, and Wigan and Bolton Health Authorities get much of their water supply from Thirlmere, a supply known to be prone to contamination by *Cryptosporidium *[6], none of the others have been implicated in outbreaks of disease. However, it would appear that the attack rates did not vary in any consistent way in relation to water source and so a waterborne hypothesis for this excess could not be proven. Analysis was restricted to Greater Manchester as analysis of all reports in the North West could be subject to confounding as a result of geographical variation in reporting behaviour, whereas the Health Authorities in Greater Manchester share a very similar notification system. The lack of an association with drinking water source was consistent with the conclusions of the case control study undertaken at the same time which also did not find an association with drinking water [9]. Nevertheless, it will be interesting to see whether the completion of an adequate water filtration plant for the Thirlmere supply which is scheduled for spring 2004 has much, if any, impact on the number of reports from Manchester.

A further explanation could be that the Manchester population experience other risk factors more commonly than the Liverpool population. Possible explanations include contact with contact with animals, visiting swimming pools and overseas travel. We do not have access to data to show whether or not people from Manchester are more exposed to these factors than people from Liverpool. However, Manchester is closer to a major National Park, The Peak District. If people from Manchester use their proximity to the Peak District to spend more time in the countryside and so are more likely to come into contact with farm animals, this could explain the difference. This would be an interesting hypothesis to test in a further study.

In addition to Greater Manchester, there are also areas of increased reporting from North Wales and from North West Lancashire. These hotspots also remain unexplained. North West Lancashire, however, receives much of its water from Thirlmere and a water source cannot be excluded. However, many cases were reported from the Fylde peninsula which only receives a small proportion of its water from Thirlmere.

## Conclusions

The use of GIS to study the spatial distribution of cases has been useful in identifying geographical variation, but not necessarily for identifying the reasons for this variation. However, initial analysis does not support the hypothesis that differences in drinking water source is the major reason for this variation. We agree with Dangendorf *et al*. [12] that GIS will contribute substantially to our understanding of the contribution of drinking water to human disease as it aids the identification of possible associations between disease and particular water supplies, provided sufficient information is collected to enable accurate location of cases.

## Methods

Consultants in Communicable Disease Control in the North West Region of England and in Wales were asked to forward details of cryptosporidium cases upon notification from the laboratory. A data collection form was completed for each case, giving the following details: name, address, postcode, date of birth, GP name, GP address and date of notification. The form was faxed or e-mailed to Communicable Disease Surveillance Centre (CDSC) – North West as soon as possible.

Enhanced surveillance for the North West of England and Wales were set up separately, North West England in mid December 2000 and Wales in February 2001. Both ran until February 2002. To check for accuracy, the data were audited every 2 months. Each CCDC was sent a list of the cases they had notified to CDSC North West in the preceding 2 months. Any cases that had not been notified were forwarded to CDSC.

The first stage in the geographical analysis was to check the 747 records for possible duplicate records. These were selected on the criteria of 2 individuals with identical names, dates of birth and postcodes being present in the database. Given that a postcode contains on average only 15 addresses the chances of these being legitimate is highly unlikely. Through this procedure 10 records were deleted from the database. Consequently 737 cases of cryptosporidiosis were identified during the period of enhanced surveillance.

The next step was to assign a grid reference to each postcode and this was achieved using the Royal Mail Postcode Address File. Eighty eight records were excluded as either, an incomplete postcode was entered into the cryptosporidiosis database or a match could not be found in the postcode address file. Therefore, in total the database was reduced to 649 cryptosporidiosis cases. These were plotted as points against a backdrop of the water supply zones for the two main water utilities. The water supply zone and water quality area boundaries were provided by the two main water utilities (United Utilities and Welsh Water). A "water supply zone" is an area designated by a water undertaker providing water to the residences of not more than 50,000 people. In general the source is consistent across a particular zone.

Using the GIS each case was also assigned its corresponding water supply zone and the number of cases in each WSZ was divided by the population, based upon data supplied by the two water utilities, to produce the attack rate maps. The analysis was undertaken in ArcGIS 8.1 using point in polygon techniques [13].

## Authors' contributions

PRH was lead on study design, did the statistical analyses and co-wrote the paper.

SH co-designed the study and co-wrote the paper and undertook most of the data collection.

QS co-designed the study and co-wrote the paper.

SW co-designed the study and co-wrote the paper.

IL undertook the geographical analyses and co-wrote the paper.

KO co-designed the study, co-wrote the paper and obtained data on the water distribution.

RC co-designed the study and co-wrote the paper.
